# Atopic Disease Prevalence by Nativity, Citizenship, and Years of Residence in the United States

**DOI:** 10.1159/drm/ackag003

**Published:** 2026-07-30

**Authors:** Vineeth R. Vaidyula, Mohnish Alishala, Helga L. Karadottir, Benjamin Ungar, Nicholas Gulati, Jonas A. Adalsteinsson

**Affiliations:** a Department of Dermatology, Icahn School of Medicine at Mount Sinai, New York, NY, USA; b Physicians for Human Rights Student Advisory Board, New York, NY, USA; c David Geffen School of Medicine at University of California Los Angeles, Los Angeles, CA, USA; d Department of Dermatology, Landspitali University Hospital, Reykjavik, Iceland; e Faculty of Medicine, University of Iceland, Reykjavik, Iceland

**Keywords:** Atopic dermatitis, Asthma, Allergic rhinitis, Immigrant, Acculturation

## Abstract

**Introduction:**

Although atopic disease, including atopic dermatitis (AD), asthma, and allergic rhinitis, is less prevalent among immigrants than natives in developed nations, its prevalence is poorly characterized among US immigrant adults. In this study, we investigated the individual and joint effects of nativity, citizenship, and duration of US residence on the prevalence of atopic disease among US adults.

**Methods:**

We analyzed 25,597 US-born and 4,714 immigrant sample adults from the 2024 National Health Interview Survey. We generated weighted prevalence estimates representative of the US and fitted logistic regression models. Models were adjusted for sociodemographics, health behaviors, and healthcare access.

**Results:**

Compared to US-born individuals, immigrants reported less AD (weighted prevalence: 4.5% vs. 8.5%; aOR: 0.56, 95% CI: [0.45–0.70]). Noncitizens also reported less AD (aOR: 0.38 [0.28–0.52]) than citizens. Moreover, compared to US-born participants, immigrant noncitizens (2.5%; aOR: 0.33 [0.23–0.45]) reported less AD than immigrant citizens (6.2%; aOR: 0.68 [0.54–0.87]). Compared to US-born participants, immigrants residing <15 years in the US (4.2%; aOR: 0.47 [0.32–0.69]) reported less AD than immigrants with ≥15 years of residence (4.7%; aOR: 0.60 [0.48–0.75]). In joint stratification analysis, AD prevalence increased progressively from recent immigrant noncitizens (2.7%; aOR: 0.30 [0.19–0.45]) to long-term immigrant citizens (5.7%; aOR: 0.64 [0.51–0.81]), and ultimately US-born citizens (8.5%; reference level), suggesting a dose-dependent relationship between acculturation and AD risk. Results were similar for asthma, allergic rhinitis, and overall atopic disease.

**Conclusion:**

Our results demonstrate a dose-dependent relationship between acculturation and atopic disease risk in the US. Early-life environmental exposures abroad may promote immune profiles associated with reduced atopic disease risk. Longer US residence may erode the protective effect of immigration via immigrants’ gradual adoption of US-specific health behaviors, including pro-inflammatory diets and obesity, increasing atopic disease risk. Citizenship demonstrated greater differences in outcomes than duration of US residence, suggesting it may capture additional factors influencing variation in atopic disease and merits further study. Study limitations include self-reported outcomes and cross-sectional design.

## Introduction

Atopic disease is less prevalent among immigrants than natives in developed nations [1]. However, the individual and joint effects of nativity, citizenship, and duration of US residence on atopic disease prevalence among adults remain uncharacterized [2]. This study aimed to investigate these effects, independent of sociodemographic factors.

## Methods

This study included sample adults from the 2024 National Health Interview Survey (NHIS) who identified as US-born or not US-born (immigrant) [3]. Atopic dermatitis (AD), asthma, and allergic rhinitis were identified based on self-reported active symptoms and prior physician diagnosis (online suppl. Table 1; for all online suppl. material, see https://doi.org/10.1159/drm/ackag003). Analyses accounted for the NHIS complex sampling design to produce US-representative prevalence estimates and subpopulation variance. Logistic regression evaluated differences in atopic disease between US-born and immigrant participants. Immigrant participants were further stratified by citizenship, years of US residence, and a combined citizenship-years of US residence variable to assess how these characteristics affect the prevalence of atopic disease. Covariates included age, sex, race/ethnicity, region, urbanicity, education, income, insurance, smoking, body mass index, healthcare visit frequency, survey language, and language at the doctor's office. Analyses were conducted using *R*, version 4.5.1.

## Results

Overall, 25,597 US-born and 4,714 immigrants were included (Table 1). In multivariable-adjusted analyses, immigrants were less likely than US-born individuals to report AD (weighted prevalence: 4.5% vs 8.5%; aOR: 0.56, 95% CI: [0.45–0.70]), asthma (4.4% vs 9.8%; aOR: 0.52 [0.41–0.65]), allergic rhinitis (14.9% vs 28.1%; aOR: 0.63 [0.56–0.71]), and overall atopic disease (AD or asthma or allergic rhinitis, 19.7% vs. 35.8%; aOR: 0.57 [0.51–0.64]) (Table 2). Compared to citizens, noncitizens also reported less AD (aOR: 0.38 [0.28–0.52]), asthma (aOR: 0.37 [0.26–0.54]), allergic rhinitis (aOR: 0.55 [0.45–0.68]), and overall atopic disease (aOR: 0.47 [0.39–0.56]).

**Table 1.** Demographic characteristics of 2024 National Health Interview Survey participants, by nativity, nativity/citizenship, and nativity/years of US residence

Demographic characteristic	US-born (citizen) (*n* = 25,597)	Any immigrant (*n* = 4,714)	Immigrant (*n* = 4,714)			
			citizen (*n* = 2,776)	noncitizen (*n* = 1,938)	<15 years in US (*n* = 1,362)	≥15 years in US (*n* = 3,352)
Age						
18–44	46.2 (0.9)	44.6 (1.9)	31.5 (2.3)	60.6 (2.7)	77.0 (2.8)	30.2 (2.0)
45–64	29.8 (0.7)	37.3 (1.7)	41.4 (2.3)	32.3 (2.6)	19.6 (2.6)	45.2 (2.0)
65+	24.1 (0.7)	18.1 (1.3)	27.1 (2.0)	7.1 (1.4)	3.4 (1.1)	24.7 (1.8)
Sex						
Female	50.9 (0.8)	51.6 (1.8)	53.8 (2.3)	48.8 (2.8)	49.3 (3.3)	52.6 (2.1)
Male	49.1 (0.8)	48.4 (1.8)	46.2 (2.3)	51.2 (2.8)	50.7 (3.3)	47.4 (2.1)
Race/ethnicity						
White	72.3 (1.3)	17.0 (1.4)	22.2 (2.0)	10.5 (1.7)	15.4 (2.3)	17.7 (1.7)
Hispanic	11.0 (0.8)	46.0 (2.7)	33.6 (3.0)	61.3 (3.2)	42.7 (4.1)	47.5 (2.9)
Black	11.9 (0.9)	10.1 (1.4)	12.5 (1.9)	7.2 (1.7)	13.9 (2.7)	8.5 (1.5)
Asian	1.7 (0.3)	25.3 (2.2)	29.6 (2.7)	20.1 (2.5)	26.9 (3.4)	24.6 (2.3)
AIAN	0.8 (0.2)	0.2 (0.2)	0.2 (0.3)	0.1 (0.1)	0.1 (0.3)	0.2 (0.2)
Other	2.4 (0.3)	1.4 (0.4)	2.0 (0.6)	0.7 (0.5)	1.0 (0.6)	1.6 (0.5)
Region						
Northeast	16.2 (1.1)	20.0 (2.5)	21.7 (2.8)	17.9 (3.1)	20.6 (3.8)	19.8 (2.6)
Central/Midwest	22.8 (1.1)	11.3 (1.7)	10.9 (1.7)	11.8 (2.4)	13.1 (2.4)	10.5 (1.9)
South	39.1 (1.5)	37.4 (3.2)	35.7 (3.5)	39.4 (4.2)	41.3 (4.4)	35.6 (3.5)
West	21.8 (1.3)	31.4 (2.8)	31.7 (3.0)	30.9 (3.7)	25.1 (3.6)	34.1 (3.2)
Urbanicity						
Urban	83.3 (1.4)	95.8 (1.3)	96.6 (0.9)	94.9 (2.1)	97.2 (1.0)	95.2 (1.6)
Rural	16.7 (1.4)	4.2 (1.3)	3.4 (0.9)	5.1 (2.1)	2.8 (1.0)	4.8 (1.6)
Education						
Less than HS	7.3 (0.5)	21.6 (1.9)	13.2 (1.8)	31.9 (2.9)	17.2 (2.8)	23.5 (2.2)
HS dipl./GED	27.5 (0.8)	23.8 (1.6)	21.6 (1.9)	26.4 (2.5)	22.3 (2.9)	24.4 (1.8)
Some college	17.1 (0.6)	10.4 (1.1)	12.8 (1.6)	7.4 (1.4)	11.0 (2.3)	10.1 (1.2)
BS degree	48.1 (1.0)	44.3 (2.0)	52.4 (2.4)	34.2 (2.8)	49.5 (3.6)	42.0 (2.3)
Income						
<200 times FPL	24.4 (0.9)	37.8 (2.1)	26.3 (2.3)	52.0 (3.0)	46.4 (3.5)	34.0 (2.5)
≥200 times FPL	75.6 (0.9)	62.2 (2.1)	73.7 (2.3)	48.0 (3.0)	53.6 (3.5)	66.0 (2.5)
Insurance						
Covered	93.2 (0.5)	82.1 (1.6)	93.6 (1.2)	67.9 (2.7)	75.7 (3.1)	84.9 (1.7)
Not covered	6.8 (0.5)	17.9 (1.6)	6.4 (1.2)	32.1 (2.7)	24.3 (3.1)	15.1 (1.7)
Smoking^a^						
<100 cigarettes	65.1 (0.8)	78.2 (1.4)	77.3 (1.7)	79.2 (2.2)	81.0 (2.4)	76.9 (1.7)
≥100 cigarettes	34.9 (0.8)	21.8 (1.4)	22.7 (1.7)	20.8 (2.2)	19.0 (2.4)	23.1 (1.7)
BMI						
<30	64.8 (0.8)	73.3 (1.7)	75.3 (2.0)	70.8 (2.5)	78.2 (2.7)	71.1 (1.9)
≥30	35.2 (0.8)	26.7 (1.7)	24.7 (2.0)	29.2 (2.5)	21.8 (2.7)	28.9 (1.9)
Healthcare visit^b^						
At least one	86.3 (0.6)	80.7 (1.5)	87.3 (1.6)	72.5 (2.5)	73.3 (2.9)	84.0 (1.6)
None	13.7 (0.6)	19.3 (1.5)	12.7 (1.6)	27.5 (2.5)	26.7 (2.9)	16.0 (1.6)
Survey language						
English	99.1 (0.2)	71.3 (2.6)	84.2 (2.3)	55.5 (3.6)	64.9 (4.1)	74.2 (2.7)
Some or no Eng.	0.9 (0.2)	28.7 (2.6)	15.8 (2.3)	44.5 (3.6)	35.1 (4.1)	25.8 (2.7)
Language at doctor’s office						
English	99.6 (0.1)	68.1 (2.3)	81.3 (2.2)	51.9 (3.4)	63.3 (3.9)	70.2 (2.5)
Other language	0.4 (0.1)	31.9 (2.3)	18.7 (2.2)	48.1 (3.4)	36.7 (3.9)	29.8 (2.5)

All data presented as weighted prevalence % (margin of error at 95% confidence).

*AIAN*, American Indian and Alaskan Native; *HS*, high school; *dipl.*, diploma; *BS*, Bachelor’s; *FPL*, federal poverty level; *BMI*, body mass index; *Eng.*, English.

^a^ Number of lifetime cigarettes.

^b^ Number of healthcare visits in the past 1 year.

**Table 2.** Prevalence and odds of atopic dermatitis, asthma, and allergic rhinitis in the US, by nativity, nativity/citizenship, and nativity/years of US residence

	US-born (citizen) (*n* = 25,597)	Any immigrant (*n* = 4,714)	Immigrant (*n* = 4,714)			
			citizen (*n* = 2,776)	noncitizen (*n* = 1,938)	<15 years in US (*n* = 1,362)	≥15 years in US (*n* = 3,352)
Atopic dermatitis						
Weighted prevalence	8.5 (0.4)	4.5 (0.7)	6.2 (1.2)	2.5 (0.7)	4.2 (1.3)	4.7 (0.9)
Crude OR model	Reference	**0.51 (0.43–0.60)**	**0.70 (0.57–0.87)**	**0.27 (0.21–0.36)**	**0.47 (0.34–0.65)**	**0.52 (0.43–0.64)**
Adjusted OR model 1^a^	Reference	**0.45 (0.37–0.56)**	**0.63 (0.50–0.79)**	**0.25 (0.18–0.34)**	**0.37 (0.26–0.54)**	**0.50 (0.40–0.62)**
Adjusted OR model 2^b^	Reference	**0.56 (0.45–0.70)**	**0.68 (0.54–0.87)**	**0.33 (0.23–0.45)**	**0.47 (0.32–0.69)**	**0.60 (0.48–0.75)**
Asthma						
Weighted prevalence	9.8 (0.5)	4.4 (0.7)	5.7 (1.0)	2.9 (0.9)	2.3 (0.9)	5.4 (0.9)
Crude OR model	Reference	**0.43 (0.36–0.51)**	**0.56 (0.46–0.68)**	**0.27 (0.20–0.38)**	**0.22 (0.14–0.33)**	**0.53 (0.44–0.63)**
Adjusted OR model 1^a^	Reference	**0.46 (0.38–0.56)**	**0.60 (0.48–0.74)**	**0.30 (0.21–0.41)**	**0.23 (0.15–0.35)**	**0.57 (0.47–0.70)**
Adjusted OR model 2^b^	Reference	**0.52 (0.41–0.65)**	**0.63 (0.50–0.79)**	**0.31 (0.21–0.46)**	**0.26 (0.17–0.42)**	**0.62 (0.49–0.78)**
Allergic rhinitis						
Weighted prevalence	28.1 (0.8)	14.9 (1.2)	18.3 (1.8)	10.7 (1.7)	10.3 (1.9)	16.9 (1.5)
Crude OR model	Reference	**0.45 (0.41–0.50)**	**0.57 (0.51–0.65)**	**0.31 (0.26–0.37)**	**0.29 (0.24–0.36)**	**0.52 (0.47–0.58)**
Adjusted OR model 1^a^	Reference	**0.54 (0.48–0.61)**	**0.67 (0.59–0.76)**	**0.39 (0.32–0.47)**	**0.36 (0.29–0.45)**	**0.63 (0.56–0.72)**
Adjusted OR model 2^b^	Reference	**0.63 (0.56–0.71)**	**0.70 (0.61–0.80)**	**0.48 (0.39–0.59)**	**0.41 (0.33–0.52)**	**0.72 (0.63–0.82)**
Any atopic disease^c^						
Weighted prevalence	35.8 (0.8)	19.7 (1.3)	24.6 (2.0)	13.8 (1.8)	14.7 (2.1)	22.0 (1.7)
Crude OR model	Reference	**0.44 (0.40–0.48)**	**0.58 (0.52–0.65)**	**0.29 (0.25–0.34)**	**0.31 (0.26–0.36)**	**0.51 (0.46–0.56)**
Adjusted OR model 1^a^	Reference	**0.49 (0.44–0.55)**	**0.63 (0.56–0.71)**	**0.33 (0.28–0.39)**	**0.34 (0.28–0.41)**	**0.57 (0.51–0.64)**
Adjusted OR model 2^b^	Reference	**0.57 (0.51–0.64)**	**0.67 (0.59–0.76)**	**0.40 (0.33–0.48)**	**0.40 (0.33–0.48)**	**0.65 (0.57–0.73)**

Prevalence data presented as weighted prevalence % (margin of error at 95% confidence). *OR*, odds ratio. Odds ratio data presented as odds ratio (95% confidence interval). Bold values indicate significance.

^a^ Adjusted for age, sex, race/ethnicity.

^b^ Adjusted for age, sex, race/ethnicity, region, urbanicity, education, income, insurance, smoking, body mass index, healthcare visit in the past year, survey language, and language at the doctor’s office.

c Includes atopic dermatitis or asthma or allergic rhinitis.

When immigrants were stratified separately by citizenship and duration of US residence and compared with US-born participants, immigrant noncitizens (2.5%; aOR: 0.33 [0.23–0.45]) reported less AD than immigrant citizens (6.2%; aOR: 0.68 [0.54–0.87]), and immigrants residing <15 years in the USA (4.2%; aOR: 0.47 [0.32–0.69]) reported less AD than immigrants residing ≥15 years in the USA (4.7%; aOR: 0.60 [0.48–0.75]). Results were similar for asthma, allergic rhinitis, and overall atopic disease (Table 2).

When immigrants were stratified jointly by citizenship and duration of US residence and compared with US-born participants, immigrant noncitizens with <15 years of US residence had the lowest odds of AD (2.7%; aOR: 0.30 [0.19–0.45]), whereas immigrant citizens with ≥15 years of US residence had the highest odds of AD (5.7%; aOR: 0.64 [0.51–0.81]). Trends were similar for asthma, allergic rhinitis, and overall atopic disease (Table 3).

**Table 3.** Prevalence and odds of atopic dermatitis, asthma, and allergic rhinitis in the US, by nativity/citizenship/years of US residence

	US-born (citizen)	Immigrant (*n* = 4714)			
		citizen	citizen	noncitizen	noncitizen
	(*n* = 25597)	<15 years in US (*n* = 305)	≥15 years in US (*n* = 2471)	<15 years in US (*n* = 1057)	≥15 years in US (*n* = 881)
Atopic dermatitis					
Weighted prevalence	8.5 (0.4)	9.2 (4.4)	5.7 (1.1)	2.7 (1.0)	2.3 (0.9)
Crude OR model	Reference	1.08 (0.64–1.82)	**0.65 (0.52–0.80)**	**0.29 (0.20–0.43)**	**0.25 (0.17–0.38)**
Adjusted OR model 1^a^	Reference	0.83 (0.48–1.43)	**0.59 (0.47–0.74)**	**0.23 (0.15–0.36)**	**0.27 (0.17–0.41)**
Adjusted OR model 2^b^	Reference	0.89 (0.52–1.53)	**0.64 (0.51–0.81)**	**0.30 (0.19–0.45)**	**0.38 (0.24–0.60)**
Asthma					
Weighted prevalence	9.8 (0.5)	3.6 (2.5)	6.0 (1.0)	1.9 (0.9)	4.0 (1.5)
Crude OR model	Reference	**0.35 (0.17–0.71)**	**0.59 (0.49–0.72)**	**0.18 (0.11–0.29)**	**0.38 (0.26–0.58)**
Adjusted OR model 1^a^	Reference	**0.36 (0.17–0.73)**	**0.64 (0.51–0.80)**	**0.19 (0.11–0.32)**	**0.42 (0.28–0.62)**
Adjusted OR model 2^b^	Reference	**0.38 (0.18–0.79)**	**0.67 (0.54–0.85)**	**0.21 (0.12–0.36)**	**0.43 (0.27–0.68)**
Allergic rhinitis					
Weighted prevalence	28.1 (0.8)	13.3 (4.6)	19.0 (1.9)	9.4 (2.1)	12.2 (2.7)
Crude OR model	Reference	**0.39 (0.27–0.58)**	**0.60 (0.53–0.68)**	**0.27 (0.21–0.34)**	**0.36 (0.28–0.46)**
Adjusted OR model 1^a^	Reference	**0.45 (0.30–0.67)**	**0.70 (0.62–0.80)**	**0.33 (0.25–0.43)**	**0.47 (0.36–0.61)**
Adjusted OR model 2^b^	Reference	**0.46 (0.31–0.69)**	**0.74 (0.65–0.85)**	**0.39 (0.29–0.51)**	**0.61 (0.46–0.82)**
Any atopic disease^c^					
Weighted prevalence	35.8 (0.8)	21.5 (5.5)	25.0 (2.1)	12.6 (2.3)	15.2 (2.8)
Crude OR model	Reference	**0.49 (0.36–0.68)**	**0.60 (0.53–0.67)**	**0.26 (0.21–0.32)**	**0.32 (0.26–0.40)**
Adjusted OR model 1^a^	Reference	**0.52 (0.37–0.72)**	**0.65 (0.58–0.74)**	**0.29 (0.23–0.36)**	**0.38 (0.30–0.48)**
Adjusted OR model 2^b^	Reference	**0.54 (0.38–0.75)**	**0.69 (0.61–0.79)**	**0.33 (0.26–0.42)**	**0.48 (0.38–0.62)**

Prevalence data presented as weighted prevalence % (margin of error at 95% confidence). *OR*, odds ratio. Odds ratio data presented as odds ratio (95% confidence interval). Bold values indicate significance.

^a^ Adjusted for age, sex, race/ethnicity.

^b^ Adjusted for age, sex, race/ethnicity, region, urbanicity, education, income, insurance, smoking, body mass index, healthcare visit in the past year, survey language, and language at the doctor’s office.

c Includes atopic dermatitis or asthma or allergic rhinitis.

## Discussion

The odds of AD, asthma, allergic rhinitis, and overall atopic disease increased progressively from recent immigrant noncitizens to long-term immigrant citizens, and ultimately US-born citizens, suggesting a dose-dependent relationship between acculturation and atopic disease risk. This finding aligns with previous observations suggesting that early-life environmental exposures abroad may promote immune profiles associated with reduced atopic disease risk [4]. Additionally, longer US residence may erode the protective effect of immigration via gradual adoption of US-specific health behaviors, including pro-inflammatory diets and obesity, increasing atopic disease risk [4]. Noncitizens had lower odds of atopic disease than US citizens, which may reflect restricted access to public benefits (including Medicaid) [5], leading to reduced healthcare utilization and potential underdiagnosis of disease; notably, 32.1% of immigrant noncitizens were uninsured and 27.5% had no healthcare visit in the past year, compared with 6.4% and 12.7% of immigrant citizens and 6.8% and 13.7% of US-born citizens (Table 1). Study limitations include self-reported outcomes and cross-sectional study design, which precludes causal inference. Citizenship, which accounted for larger differences in outcomes among immigrants than duration of US residence, may capture additional factors driving variation in atopic disease, warranting further investigation. Overall, the observed gradient in atopic disease prevalence across acculturation subgroups demonstrates heterogeneity in atopic disease burden among immigrants and warrants further investigation into the environmental, behavioral, and healthcare-access-related factors underlying these patterns.

## Statement of Ethics

Ethical approval and consent were not required as this study was based on publicly available data.

## Conflict of Interest Statement

B.U. is an employee of Mount Sinai and has received research funds (grants paid to the institution) from: Bristol Myers Squibb, Incyte, Rapt Therapeutics, Pfizer, and Sanofi. He is also a consultant for AbbVie, Arcutis Biotherapeutics, Apogee Therapeutics, Bristol Myers Squibb, Botanix Pharmaceuticals, Castle Biosciences, Ebla Holdco, Fresenius Kabi, Galderma, Incyte, J&J, Leo Pharma, Lilly, Nektar Therapeutics, Pfizer, Primus Pharmaceuticals, Sanofi, Sun Pharma, UCB, Veradermics, VRG Therapeutics. N.G. is an employee of Mount Sinai and is a consultant for the following companies: Almirall, Daiichi Sankyo, and Primus Pharmaceuticals. V.R.V., M.A., H.L.K., and J.A.A. report no conflicts of interest.

## Funding Sources

This study was not supported by any sponsor or funder.

## Author Contributions

V.R.V. contributed to conceptualization, methodology, formal analysis and investigation, writing – original draft preparation, writing – review and editing, and resources. M.A. contributed to writing – original draft preparation and writing – review and editing. H.L.K., B.U., and N.G. contributed to writing – review and editing. J.A.A. contributed to conceptualization, writing – review and editing, and supervision. All authors reviewed the manuscript.

## Data Availability

The data that support these findings are publicly available on the IPUMS Health Surveys website: https://healthsurveys.ipums.org/. Further inquiries can be directed to the corresponding author.
